# The Modified Surface Killing Assay Distinguishes between Protective and Nonprotective Antibodies to PspA

**DOI:** 10.1128/mSphere.00589-19

**Published:** 2019-12-11

**Authors:** Kristopher R. Genschmer, Cintia F. M. Vadesilho, Larry S. McDaniel, Sang-Sang Park, Yvette Hale, Eliane N. Miyaji, David E. Briles

**Affiliations:** a Department of Microbiology, University of Alabama at Birmingham, Birmingham, Alabama, USA; b Laboratório de Bacteriologia, Instituto Butantan, Sao Paulo, Sao Paulo, Brazil; c Department of Microbiology and Immunology, University of Mississippi Medical Center, Jackson, Mississippi, USA; d Department of Genetics, University of Alabama at Birmingham, Birmingham, Alabama, USA; e Department of Pediatrics, University of Alabama at Birmingham, Birmingham, Alabama, USA; U.S. Food and Drug Administration

**Keywords:** PspA, modified surface killing assay, pneumococcal surface protein A

## Abstract

Pneumococcal surface protein A (PspA) elicits antibody protective against lethal challenge by Streptococcus pneumoniae and is a candidate noncapsular antigen for inclusion in vaccines. Evaluation of immunity to PspA in human trials would be greatly facilitated by an *in vitro* functional assay able to distinguish protective from nonprotective antibodies to PspA. Mouse monoclonal antibodies (MAbs) to PspA can mediate killing by human granulocytes in the modified surface killing assay (MSKA). To determine if the MSKA can distinguish between protective and nonprotective MAbs, we examined seven MAbs to PspA. All bound recombinant PspA, as detected by enzyme-linked immunosorbent assay and Western blotting; four gave strong passive protection against fatal challenge, two were nonprotective, and the seventh one only delayed death. The four that were able to provide strong passive protection were also most able to enhance killing in the MSKA, the two that were not protective in mice were not effective in the MSKA, and the MAb that was only weakly protective in mice was weakly effective in the MSKA (*P < *0.001). One of the four most protective MAbs tested reacted to the proline-rich domain of PspA. Two of the other most protective MAbs and the weakly protective MAb reacted with a fragment from PspA’s α-helical domain (αHD), containing amino acids (aa) 148 to 247 from the N terminus of PspA. The fourth highly protective MAb recognized none of the overlapping 81- or 100-aa fragments of PspA. The two nonprotective MAbs recognized a more N-terminal αHD fragment (aa 48 to 147).

**IMPORTANCE** The most important finding of this study is that the MSKA can be used as an *in vitro* functional assay. Such an assay will be critical for the development of PspA-containing vaccines. The other important findings relate to the locations and nature of the protection-eliciting epitopes of PspA. There are limited prior data on the locations of protection-eliciting PspA epitopes, but those data along with the data presented here make it clear that there is not a single epitope or domain of PspA that can elicit protective antibody and there exists at least one region of the αHD which seldom elicits protective antibody. Moreover, these data, in concert with prior data, strongly make the case that protective epitopes in the αHD are highly conformational (≥100-amino-acid fragments of the αHD are required), whereas at least some protection-eliciting epitopes in the proline-rich domain are encoded by ≤15-amino-acid sequences.

## Abstract (precis)

The most important finding of this study is that the MSKA can be used as an *in vitro* functional assay. Such an assay will be critical for the development of PspA-containing vaccines. The other important findings relate to the locations and nature of the protection-eliciting epitopes of PspA. There are limited prior data on the locations of protection-eliciting PspA epitopes, but those data along with the data presented here make it clear that there is not a single epitope or domain of PspA that can elicit protective antibody and there exists at least one region of the αHD which seldom elicits protective antibody. Moreover, these data, in concert with prior data, strongly make the case that protective epitopes in the αHD are highly conformational (≥100-amino-acid fragments of the αHD are required), whereas at least some protection-eliciting epitopes in the proline-rich domain are encoded by ≤15-amino-acid sequences.

## INTRODUCTION

Pneumococcal surface protein A (PspA) is found on the surface of virtually all Streptococcus pneumoniae strains (1, 2). It is necessary for the full virulence of the pneumococci in systemic mouse infections (3, 4) and also plays a role in nasopharyngeal colonization (5), otitis media (6), and lung infection (S.-S. Park and D. E. Briles, unpublished results). Immunity to PspA has also been shown to protect against systemic infection (7–9), pneumonia (10), carriage (11–13), and otitis media (14). PspA has been shown to be an immunogenic carrier for polysaccharides (13). Immunization of humans with PspA elicits antibody that can passively protect mice against pneumococcal infection (15). PspA is probably the most common choline-binding protein expressed on the surface of pneumococci (16), and antibody to PspA can enhance complement deposition and phagocytosis by immune cells (17–20). The presence of PspA on the bacterial surface enhances the ability of pneumococci to inhibit complement deposition (4, 21), protects the bacteria from killing by apolactoferrin (22), and also protects against complement-independent opsonophagocytic killing of pneumococci (23) and *in vitro* has been shown to protect against pneumococcal killing by neutrophil extracellular nets (24) . PspA is considered a potential vaccine candidate for use in humans (25–27).

Mature PspA is composed of three major domains: its N-terminal α-helical domain (composed of from 300 to 400 aa, which are in an antiparallel coiled-coil structure [28–31]), the 65- to 100-aa proline-rich domain (PRD) (28, 32, 33), and finally the C terminus containing a roughly 200-aa choline-binding domain. Both the αHD and PRD are able to elicit protective immunity in mice (10, 11, 15, 34–37). There are two regions of the αHD that appear to elicit protection. One is from amino acids 51 to 100 within the N-terminal region of αHD, and the other is within the αHD’s roughly 100 C-terminal amino acids (27, 38, 39).

The current pneumococcal vaccine used in the United States is a 13-valent pneumococcal conjugate vaccine (PCV13), consisting largely of the 13 serotypes most clinically relevant in the United States in 2000. While this vaccine is highly successful at protecting against bacteremic disease (40), it is less protective against pneumococcal meningitis and nonbacteremic pneumococcal pneumonia (41–43) and is not protective against total pneumococcal colonization (44). The conjugate vaccine is relatively expensive and has been shown to select for serotype replacement by nonvaccine serotypes in colonization (43) and to a lesser degree in invasive disease (45, 46). A protein-based vaccine, if developed, might be much easier to produce than the conjugate vaccine, will likely cover strains of diverse capsular types (25, 47), and would, it is hoped, be more affordable, especially in the developing world.

A big hurdle in the development of protein antigens, such as PspA, for use in pneumococcal vaccines is that the needed vaccine trials are hampered by the lack of established *in vitro* surrogates for the protection elicited by protein antigens. For antibodies to pneumococcal capsular polysaccharides, an opsonophagocytic killing assay (OPKA) that serves as an *in vitro* surrogate that evaluates the ability of potential conjugate vaccines to protect has been developed (18, 48–50). However, this OPKA, developed for polysaccharides, does not readily detect protective antibody to other pneumococcal antigens (51), although such antibodies are clearly protective in mouse models. OPKA fails to detect the protection elicited by other antigens probably because an OPKA design that excludes the detection of background protection was settled on. We suspect that this background protection is likely the result of antibodies to other antigens of pneumococci (25, 47, 52). There have been several demonstrations of functional *in vitro* assays that detect the protection mediated by antibodies to pneumococcal proteins (23, 51–53). One such assay, the modified surface killing assay (MSKA), detects the *in vitro* killing of pneumococci mediated by monoclonal antibodies (MAbs) to PspA (23).

For an *in vitro* assay to have the potential to predict optimal *in vivo* protection and, thus, be potentially useful in the evaluation of the dose-response and in vaccine composition studies in humans, the assay should be able to distinguish between antibodies to the target antigen that are highly protective, weakly protective, and nonprotective against pneumococcal disease. To determine if the MSKA could distinguish between protective and nonprotective antibodies to PspA, we examined seven MAbs. The MAbs examined included four new antibodies raised against the recombinant αHD of the PspA of strain Rx1 (PspA/Rx1; family 1, clade 2) as well as an MAb raised against the nonproline block (NPB) of the PRD of the Rx1 PspA (37). We also included two MAbs to PspA that we reported on previously (23). Of these MAbs, four were strongly passively protective, one was weakly protective, and two provided no passive protection. In the present study, we used seven overlapping 81- to 100-aa fragments of PspA and overlapping 15-aa peptides in an attempt to map the epitopes recognized by the MAb.

## RESULTS

### Close similarity of PspA/Rx1, PspA/A66.1, and PspA/WU2.

The structural similarities between the PspAs of strains A66.1 (PspA/A66.1), Rx1 (PspA/Rx1), and WU2 (PspA/WU2) are relevant to the interpretation of the results presented in this paper and prior studies. The PspAs of strains A66, A66.1, and WU2 are all identical in their αHDs (see Text S1 in the supplemental material). These three PspAs are also identical to PspA/Rx1 in the 116 C-terminal amino acids of the αHD, which includes the clade-defining regions (CDRs) of their αHDs. PspA/A66.1 and PspA/WU2 were 60% identical to PspA/Rx1 in the region of the mature sequence from aa 18 to 148 (Text S1). These findings are important because all three PspAs are used in these studies.

10.1128/mSphere.00589-19.2 TEXT S1 Rationale for the pneumococcal strains and PspA sequences used. Download Text S1, DOCX file, 0.01 MB. Copyright © 2019 Genschmer et al. 2019 Genschmer et al. This content is distributed under the terms of the Creative Commons Attribution 4.0 International license.

### Reactivity of seven MAbs with rPspA/Rx1 and 81- or 100-aa PspA/Rx1 fragments.

**FIG 1:**
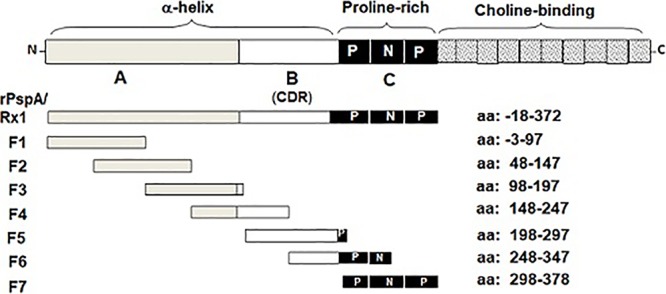
Schematic diagram of PspA/Rx1 fragments 1 to 7 (F1 to F7, respectively) (adapted from reference 28). At the top is shown the whole mature PspA molecule, containing the N-terminal αHD (including regions A and B), the proline-rich domain (C), and the choline-binding domain at the C terminus. In the center of the proline-rich domain is a nonproline block (N) surrounded by proline-rich sequences (P). The PspA leader (not shown) is removed during PspA expression by pneumococci. The B region of the αHD has been called the clade-determining region (28). Also shown are six overlapping 81- or 100-amino-acid fragments covering the α-helical and proline-rich domains. The sequence positions of the first and final amino acids of each fragment are listed at the right.

**FIG 2:**
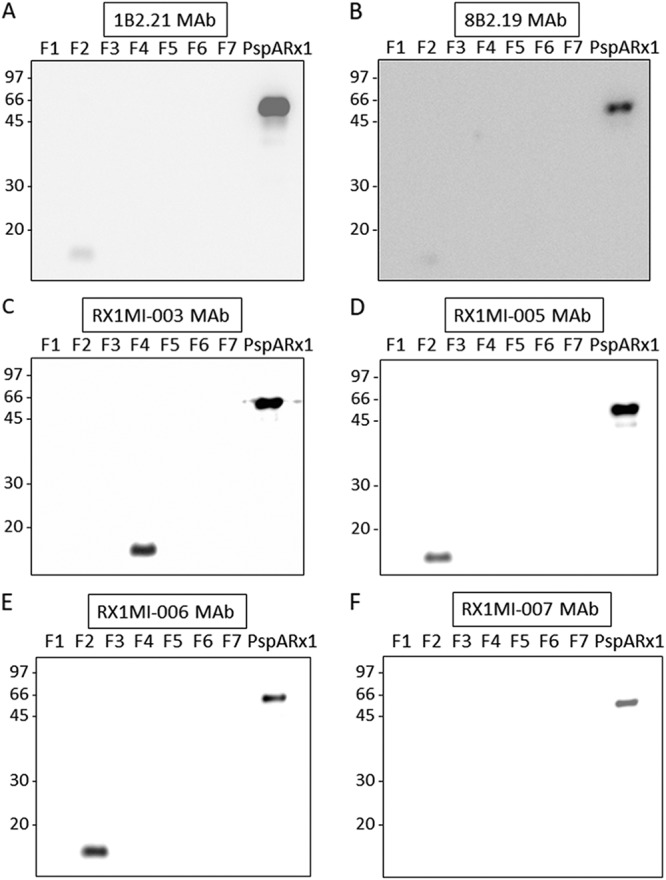
Western blotting of the reactivities of six of the seven MAbs, 1b2.21 (A), 8b2.19 (B), Rx1MI003 (C), Rx1MI005 (D), Rx1MI006 (E), and Rx1MI007 (F), with rPspA/Rx1 (aa 1 to 381) and 81- or 100-amino-acid PspA/Rx1 fragments 1 to 7 (F1 to F7, respectively, described in Fig. 1). The positions of molecular mass standards (in kilodaltons) are indicated on the left.

The recombinant PspA (rPspA)/Rx1 (aa −18 to aa 372) and the seven overlapping 81- to 100-aa fragments (six of which were 100 aa and one of which was 81 aa) of the contiguous αHD and PRD of PspA/Rx1 (Fig. 1; Table 1) were tested for reactivity with the seven MAbs by Western blotting. Each MAb reacted with rPspA/Rx1. Western blotting data for the six MAbs to αHD are shown in Fig. 2 for the anti-PRD MAb. The data for MAb PR-1A4.7 were previously published (37). MAbs Rx1MI005 and Rx1MI006 showed relatively strong reactivity with fragment 2 (Fig. 2D and E) but not with any other fragments. MAb 1b2.21 showed very faint reactivity with fragment 2 (Fig. 2A), but its reactivity with fragment 2 was judged to be negative in comparison to the reactivity of Rx1MI005 and Rx1MI006. MAb 1b2.21 did not react with any of the other fragments. MAb Rx1MI003 reacted only with fragment 4 (Fig. 2C). Although MAbs Rx1MI007 and 8b2.19 reacted strongly with rPspA/Rx1 (Fig. 2B and F), they failed to react significantly with any of the 81- to 100-amino-acid fragments.

**TABLE 1:** Reactivity of MAbs with native PspA, 81- or 100-aa peptides of PspA, and the PspA PRD

MAb	Reactivity with Rx1 and A66.1 PspAs^*a*^	Fragment of Rx1 αHD with which MAb was reactive by:		Reactivity with PRD	Reference or source for MAb
		ELISA	Western blotting		
Rx1MI005	+	2 (48–147)	2 (48–147)	−	This paper
Rx1MI006	+	2 (48–147)	2 (48–147)	−	This paper
Rx1MI007	+	None	None	−	This paper
PR-1A4.7	+	Not done^*b*^	Not done^*b*^	+	37
Rx1MI003	+	4 (148–247)	4 (148–247)	−	This paper
8b2.19	+	None	None	−	23
1b2.21	+	4 (148–247)	None	−	23

a Reactivity with rPspA was determined by dot blotting of the MAb with rPspA/Rx1 and bacterial lysates of A66.1. The αHDs of PspA/Rx1 and PspA/A66.1 PspA are both family 1, clade 2, and are 85% homologous. The proline-rich domains of the Rx1 and A66.1 PspAs are 80% homologous. The MAbs in this table are listed in the same order as in Table 2 to facilitate comparison of the MAb properties.

b The specificity of MAb PR-1A4.7 was not examined in this study, but it has previously been shown to react with the NPB of the Rx1 PRD and to react with whole PspA (37). The NPBs of PspA/Rx1 and PspA/A66.1 are identical (28, 60).

We also checked the reactivities of the six MAbs with rPspA/Rx1 by enzyme-linked immunosorbent assay (ELISA), and all reacted strongly. The strongest reactivity to rPspA/Rx1 was with MAbs Rx1MI003, -005, and -006 (Table 1; Fig. S1). By Western blotting, all six MAbs also bound rPspA/Rx1, but this time the strongest reactivities appeared to be with MAbs 1b2.21, Rx1MI003, and Rx1MI005. The small differences in binding between the MAbs to Rx1/PspA by Western blotting and the ELISAs may not be significant. The seventh MAb, MAb PR-1A4.7, which was reactive with NPB, was previously shown to bind to intact PspA on the bacterial surface (37). When the overlapping 81- to 100-aa fragments were tested for reactivity with the six MAbs by ELISA, it was observed that MAbs Rx1MI005 and Rx1MI006 bound to fragment 2, MAbs 1b2.21 and Rx1MI003 bound to fragment 4, and MAbs 8b2.19 and Rx1MI007 did not bind to any of the fragments. When the reactivities of the six MAbs to the αHD with the overlapping fragments were analyzed by Western blotting, results that were the same as those obtained by ELISA were found for all MAbs, except that MAb 1b2.21 showed no detectable reactivity with MAb fragment 4 (Table 1). This may be an indication that the epitope on fragment 4 recognized by 1b2.21 was denatured by the sodium dodecyl sulfate (SDS) used in the electrophoresis buffer used in the Western blotting procedure, whereas the epitopes of the other three MAbs survived the SDS treatment.

10.1128/mSphere.00589-19.1 FIG S1 ELISA showing the binding of individual MAbs to rRx1/PspA and our 81- to 100-amino acid fragments of PspA/Rx1. Microtitration plates were coated with the indicated proteins and incubated with the respective MAb at 0.125 μg/ml. Binding of the MAb to the proteins was detected with anti-mouse immunoglobulin conjugated with alkaline phosphatase. The results are reported as the optical density at 492 nm. The results of the assay indicate that all six MAbs bound to the rPspA/Rx1 αHD fragment. However, only four MAbs bound to any of the 81- to 100-aa overlapping fragments of the rPspA/Rx1 αHD fragment. MAbs Rx1MI005 and Rx1MI006 bound overlapping 100-aa fragment 2, and MAbs Rx1MI003 and 1b2.21 bound 100-aa fragment 4. Download FIG S1, TIF file, 0.1 MB. Copyright © 2019 Genschmer et al. 2019 Genschmer et al. This content is distributed under the terms of the Creative Commons Attribution 4.0 International license.

### Ability of MAbs to the αHD of PspA to bind to the panel of overlapping 15-aa peptides representing the αHDs of clades 1 to 5.

The six MAbs made against the αHDs were tested against the array of 15-aa peptides with 11-aa overlaps. The array covers the sequence of clade 2 PspA/Rx1. None of the MAbs to the αHDs of PspA bound to any of the 15-aa peptides (data not shown). This suggests that all of our MAbs to the αHDs most likely recognize conformational epitopes. The epitopes of these antibodies are probably dependent in large part on interactions within the antiparallel coil-coil structure of the αHD of PspA (29). This observation is consistent with earlier findings that other MAbs to the αHD bound only to PspA fragments of 100 amino acids or larger (39) and more recent findings that the protective polyclonal antisera to PspA bound to rPspA and large 100-amino-acid fragments. The only 15-amino-acid peptides that these antisera recognized were a few near the N terminus. A 38-aa construct of the most conserved N-terminal amino acids failed to elicit protection (38).

The MAb PR-1A4.7 has previously been shown to react with the NPB of the PRD (37), which is part of PspA/Rx1. In this study, we found that PR-1A4.7 reacted with two overlapping peptides of the peptide array, which represented part of the NPB. PR-1A4.7 bound within the sequence A-R-R-S-E-E-E-Y-N-R-L. PR-1A4.7 also binds full-length Rx1/D39 PspA (37).

### Relative ability of MAbs to PspA to enhance killing by neutrophils in MSKA.

**FIG 3:**
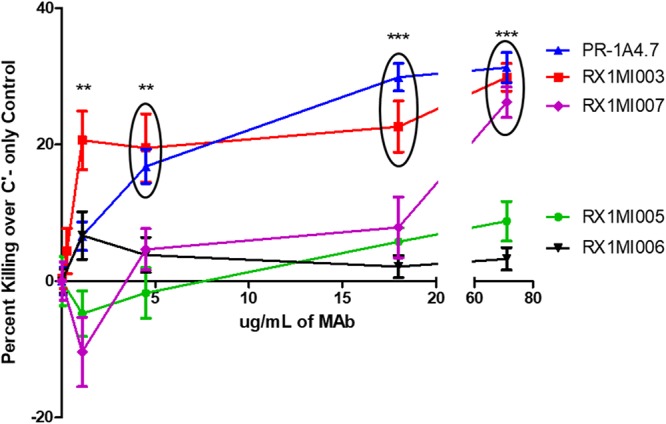
Comparative killing of type 3 strain A66.1 in the MSKA mediated by five of the seven anti-PspA MAbs, Rx1MI003, PR-1A4.7, Rx1MI007, Rx1MI006, and Rx1MI005, each of which reacted with A66.1 PspA. Significance was determined by comparing the results at each MAb concentration to the results obtained with the complement-only control. All data points within a circle are of similar significance. The PR-1A4.7 antibody at a concentration of 1.1 μg/ml had a *P *value of <0.05 but is not marked. **, *P* < 0.01; ***, *P < *0.001. All other comparisons had *P *values of *≥*0.05.

This study compared the ability of the seven MAbs to enhance the killing of type 3 pneumococcal strain A66.1 in the MSKA (Table 2). The results obtained with five of the MAbs are also shown in Fig. 3. Two of the MAbs to αHD, 1b2.21 and 8b2.19, were already known to be passively protective and to enhance the killing of A66.1 in the MSKA (23). The study also included the PRD-binding MAb PR-1A4.7, which protects against invasive disease in mice (37) but which has not been tested in the MSKA. Four new MAbs to PspA produced for this study were also examined in the MSKA, MAbs Rx1MI003, -005, -006, and -007.

**TABLE 2:** Strong correlation between ability of MAb to PspA to mediate protection in the MSKA and in passive protection *in vivo*^*a*^

MAb^*b*^	Minimum amt (μg) of MAb to give 10% killing in MSKA^*c*^^,^^*d*^	% killing by 4.5 μg^*c*^ MAb/mouse in MSKA	Median time (h) to moribund state with 5 μg MAb/mouse
Negative control^*e*^		0.0	24
Rx1MI005	>70	1.5	30
Rx1MI006	>70	3.3	42
Rx1MI007	70	5.5	141
PR-1A4.7	5	16.8	>336
Rx1MI003	1	21.5	>336
8b2.19^*f*^	0.1	22.2	>336
1b2.21^*f*^	0.1	25.7	>336

a The target strain for the MSKA and the challenge strain for the passive protection assay was capsular type 3 strain A66.1. The linear correlation coefficient (*r*) for percent killing versus the median time (in hours) to a moribund state for the seven MAbs was 0.957 (95% confidence interval, 0.731 to 0.994; *P* = 0.0007). The correlation coefficient for the minimum amount of MAb required to give 10% killing versus the median time (in hours) to a moribund state was −0.9949 (*P* < 0.0001). For both comparisons, the correlation coefficient calculated by the Spearman rank test was 0.9063 (*P *< 0.0011). Calculation of the correlation coefficients did not include the data for the negative control, which would have erroneously enhanced the *P* values. For calculations of the correlation coefficient and *P* value, values of >70 were conservatively assigned a value of 90.

b All MAbs bind to native PspA. All MAbs except PR-1A4.7 bind the PspA α-helical domain. PR-1A4.7 binds the PspA proline-rich domain.

c In the MSKA, the indicated MAbs were each tested separately in a total volume of 300 μl of reaction mixture.

d All values were rounded to one significant figure.

e The negative control for the MSKA and the passive protection assay were Ringer’s solution and NMS, respectively.

f The passive protection assay data for MAbs 8b2.19 and 1b2.21 were taken from our previous paper describing MSKA (23) and are provided for comparison with the data for the other MAbs. Both MAbs were studied with 1/10 dilutions, so the median time (in hours) to a moribund state was interpolated to values at 5 μg/ml based on data that either bridged 5 μg/ml or where one data point was 5 μg/ml (23).

PR-1A4.7 and the four new MAbs were tested for their ability to enhance killing in the MSKA. Each MAb was used in the MSKA at 71 μg/ml and at four sequential 1/4 serial dilutions. As seen in Fig. 3, the ability of Rx1MI003 to mediate the killing of A66.1 over that for the complement-only control was from a maximum of 20 to 30% killing (*P < *0.001) down to 18% killing at antibody concentrations of 1.1 μg/ml. MAb PR-1A4.7 mediated a 30% increase in killing at 71 and 18 μg/ml and continued to give statistically significant killing down to 1.1 μg/ml (Fig. 3). Rx1MI007 mediated a significant increase in killing in the MSKA at 71 μg/ml but no protection at lower concentrations (Fig. 3). MAbs Rx1MI005 and Rx1MI006 did not show any significant increase in killing of A66.1 in the MSKA over that for the complement-only control at any antibody concentration (Fig. 3).

### Passive protection studies showed that the MAbs to PspA that facilitated increased killing in MSKA also protected mice from lethal challenge.

CBA/CaHN-Btk^xid^/J (CBA/N) mice were used for the passive protection studies because they are highly susceptible to pneumococcal sepsis (54) and have been used extensively in passive protection studies in the past (15, 23). The CBA/N mice provide a particularly sensitive model of protection by antibodies to PspA since their serum invariably lacks any protective antibody to capsular or phosphocholine-bearing teichoic or lipoteichoic acids (15, 54). Control mice received 1/6 normal mouse serum (NMS) diluted in Ringer’s lactate, since this provided a concentration of total serum protein comparable to that in most of the diluted ascitic fluids used for passive protection. The MAbs were given at 5 μg/mouse so that the amount of antibody would be limiting.

**FIG 4:**
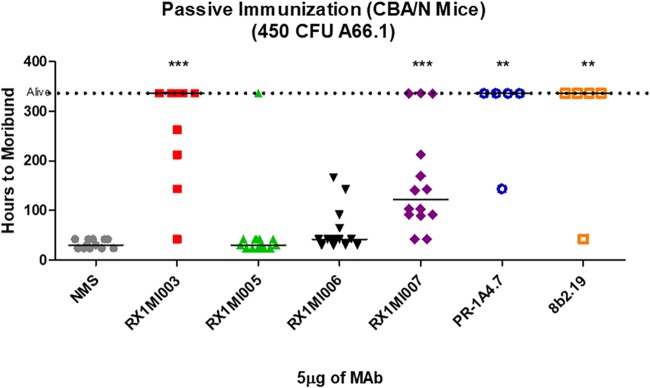
Passive immunization results obtained with six of the seven MAbs in mice, which received a lethal i.v. challenge with A66.1. CBA/N mice were passively immunized i.p. with 0.1 ml containing 5 μg of anti-clade 2 MAb Rx1MI003, Rx1MI005, Rx1MI006, Rx1MI007, PR-1A4.7, or 8b2.19. Control mice were given 0.1 ml 1/6 NMS. Four hours later the mice were challenged i.v. with 450 CFU of A66.1. The time (in hours) to a moribund state is given for each mouse. Mice given Rx1MI003, Rx1MI007, PR-1A4.7, or 8b2.19 showed a significantly greater time to a moribund state than NMS-treated control mice (**, *P* < 0.01; ***, *P < *0.001). Mice given Rx1MI005 or -006 had times to a moribund state that were not statistically different from those for the NMS-treated controls. The colors of the MAb data points correspond to the colors of the MAbs identified in the key in Fig. 3.

In this experiment, all of the CBA/N mice in the NMS-treated control group became moribund within 48 h after challenge, and the median time to a moribund state was 24 h (Table 2; Fig. 4). The two MAbs Rx1MI005 and Rx1MI006, which failed to show statistically significant protection in the MSKA (Fig. 3), were not able to show significant passive protection of mice from fatal infection.

Conversely, all MAbs that significantly enhanced killing in the MSKA (MAbs 1b2.21, 8b2.19, PR-1A4.7, Rx1MI003, and Rx1MI007) also facilitated median survival times of 141 h or greater (*P < *0.001) (Table 2; Fig. 4), and the association between the median time to a moribund state for mice treated with each MAb and the same MAb’s ability to enhance killing in the MSKA was significant (*P < *0.001) (Table 2). It should be noted that MAb Rx1MI007, which facilitated the shortest increase in survival time *in vivo* (141 h), had to be used at the highest concentration to facilitate bacterial killing in the MSKA (Fig. 3C). The protection afforded by Rx1MI007 in both the passive protection assay and the MSKA was therefore intermediate between that of the highly protective MAb and that of the NMS control. These observations strongly indicate that the MSKA is capable of identifying weakly protective antibody, a feature that could be critical for an *in vitro* assay to be useful as a good surrogate of protection. In contrast, the ELISA (Fig. S1) showed that the reactivity of each of the six MAbs against rPspA tested was equivalent, even though two of them did not exhibit any protection in mice or in the MSKA. Moreover, the amount of signal detected in the ELISA with the different MAbs also failed to correlate with differences in the ability of the different MAbs to mediate passive protection or to facilitate partial protection in the MSKA. These findings indicate that of the *in vitro* assays, the MSKA was a better indicator of passive protection in mice than either the ELISA or Western blotting.

### Relationship between protection and specificity of the MAbs.

Of the four most protective MAbs in mice or in the MSKA (Table 2), one (MAb PR-1A4.7) bound the PRD and two (MAbs Rx1MI003 and 1b2.21) bound fragment 4 (aa 148 to 247). The fourth highly protective MAb, 8b2.19, was elicited by immunization with the αHD and recognized rPspA but did not recognize any of the seven recombinant overlapping 100-aa fragments. The moderately protective MAb Rx1MI007 bound to PspA/A66 and the α-helical domain of PspA/Rx1 but failed to bind to any of the 100-amino-acid fragments. The two nonprotective MAbs, Rx1MI005 and Rx1MI006, recognized only PspA/A66 and the 47- to 148-aa fragment of PspA/Rx1. It is possible that the three MAbs that bound to PspA/A66 and rPspA/Rx1 but none of the 81- to 100-aa fragments recognized highly conformational epitopes that were not expressed on PspA fragments as small as the 100-aa overlapping fragments used here.

## DISCUSSION

### Potential of MSKA to serve as an *in vitro* functional assay to detect protective antibody to PspA.

It is well established that human and mouse antibodies to PspA can actively and passively protect mice from lethal pneumococcal challenge (9, 15, 52, 55, 56). One hurdle that must be overcome for PspA to move into efficacy trials has been the lack of an accepted *in vitro* functional assay to screen sera from immunized humans for changes in their protective potential. Such an *in vitro* correlate of protection exists for immunity to capsular polysaccharides, for which the OPKA has been accepted as a useful surrogate (48).

In the present studies, of the seven MAbs known to bind PspA/A66.1 of the challenge strain, four were highly protective against the strain in the MSKA and in a passive *in vivo* protection assay, two were nonprotective in both assays, and one showed weak protection both in the MSKA and in the passive protection assay. These results indicate that the MSKA should be useful for predicting the relative level of *in vivo* protection elicited by PspA. In contrast to the results obtained with the MSKA, the detection of antibody to rPspA by neither the Western blot assay nor the ELISA correlated with the ability of the antibody to protect against fatal infection in mice. Six MAbs were examined in the Western blot assay (Fig. 1), and the seventh was evaluated by Daniels et al. (37). All seven MAbs bound rPspA, but only four were protective. Three of the six MAbs from Fig. 1 bound to one or more of the 81- to 100-aa PspA fragments, but only two of these were protective. Moreover, another MAb was strongly protective but bound none of the 81- to 100-aa PspA fragments by ELISA. The same six MAbs were examined for reactivity with the fragments by ELISA; all six detected rPspA/Rx1, but only three were protective. Thus, assays that merely detect antibody to PspA will probably not be adequate to predict which human responses to PspA are the most protective. Further study of the MSKA is needed to see if the results presented here will hold up with immune sera and other strains of pneumococci with different PspAs.

Our present results provide encouragement that the modified OPKA that detects protection by antibody to PspA (51) might also be useful as an *in vitro* functional assay for human antibody to PspA. It should also be noted that, using a different OPKA to study antisera to 17 separate proteins, it was found that of the 17 different OPKA-positive antisera, all but 1 gave passive protection in mice (52).

### Protection-eliciting epitopes of PspA.

Since not all of the MAbs that bound to PspA of the target strain protected against pneumococci in the MSKA or in passive protection assays *in vivo*, it is possible that some regions of PspA might be more likely than others to elicit protection. We attempted to map the epitopes of the protective and nonprotective MAbs using panels of overlapping 100-amino-acid peptides and 15-amino-acid overlapping peptides, each of which covered the αHD and PRD of PspA.

Several studies have shown that more than one epitope of PspA is able to elicit protection and that the most protective antibodies to PspA are elicited by either the C- or N-terminal third of the αHD of PspA (27, 37–39, 55). Of the seven MAbs to PspA tested in this study, the only one that reacted with any of the overlapping 15-aa peptides was the MAb previously shown to react with the PRD of PspA (37). We also examined the reactivity of the five protective MAbs and two nonprotective MAbs with our seven overlapping 81- to 100-aa peptides of PspA. Of the four most protective MAbs, two (1b2.21 and Rx1MI003) mapped within amino acids 148 to 247 of the αHD. This region contains the N-terminal half of CDR. The epitope of the third strongly protective MAb (PR-1A4.7) mapped to the PRD (37). The epitope of the fourth strongly protective MAb (8b2.19) did not bind any of the 81- to 100-amino acid fragments but did react with the intact αHD fragment of rPspA. This implied that it was directed against an extremely conformationally dependent epitope in the αHD. The weakly protective MAb (Rx1MI007) also reacted with the intact αHD of PspA but not with any of the 81- to 100-amino acid fragments. The two MAbs that were reactive with PspA but that were not protective (Rx1MI005 and Rx1MI006) bound the fragment containing aa 48 to 147. Thus, in this study all protection-eliciting epitopes that could be mapped were represented in the portion of the αHD fragment including either aa 148 to 247 or the NPB.

In a prior study using a different set of larger PspA fragments and a different set of MAbs, four of five protective MAbs mapped within amino acids 192 to 260 of the α-helical domain and one mapped within amino acids 1 to 115 (39). XiR278 was one of the prior protection-eliciting MAbs mapping within aa 192 to 260. XiR278 was recently tested for its protective effects in the MSKA and in passive protection assays in mice, and it was found to be highly protective in both assays (23). This prior finding with XiR278 mapping to aa 192 to 260 (39) supports our present data showing that epitopes that can elicit protection in the MSKA are generally found between amino acids 151 and 250. The findings of this study are consistent with those of two prior studies, which showed that immunization with fragments of PspA, including the N-terminal end and the C-terminal end of the αHD of PspA, but not the middle of the αHD of PspA, elicit the best protection (27, 57).

### Evidence for the conformational nature of αHD MAb epitopes of PspA.

The fact that none of the six MAbs to the αHD of PspA that were elicited bound any of the peptides in the 15-aa overlapping PspA peptide panel strongly indicates that MAbs to the αHD detect conformational epitopes, which could not be formed by small peptides. The failure of two of the six MAbs to the αHD to react with any of the seven overlapping 81- to 100-aa fragments of PspA suggests that the amino acids required for the conformations of their epitopes are dependent on regions of the sequence that are not all found in a single PspA fragment. Moreover, each of the four MAbs that bound to any of the 81- to 100-amino acid fragments bound only one fragment, even though all of the amino acid sequence of each fragment was also found in one of the adjacent fragments attached to different amino acids. These results are consistent with those of a previous study, which showed that as the target molecules of anti-PspA MAbs got smaller than about 150 aa, antibody binding invariably got weaker or disappeared (39). Furthermore, immunization with peptides of conserved sequences in the N-terminal end of PspA did not elicit antibody reactive with PspA on the bacterial surface and did not elicit protection, even though the peptides were immunogenic (38).

The fact that protection-eliciting epitopes of the αHD of PspA are generally conformational epitopes (38, 39) is probably the result of the fact that the αHD structure is largely an antiparallel stacked α-helical coiled coil (29) in which the conformation at any point in the sequence would be expected to be dependent on amino acids in the antiparallel α-helical coils. These observations emphasize the importance of immunization with large αHD fragments and may also indicate the need for caution about the appropriate design and usefulness of recombinant PspA molecules created by stitching together fragments of the αHDs of diverse PspAs.

Most importantly, these data show that the MSKA can be used to differentiate between antibodies to PspA that bind PspA to identify those that are protective *in vivo*.

## MATERIALS AND METHODS

### Bacterial strains.

The highly virulent type 3 pneumococcal strain A66.1 (58, 59) was grown in Todd-Hewitt broth containing 0.5% added yeast extract (THY) at 37°C until it reached an optical density at 600 nm of 0.40 to 0.45. The bacterial stock was then washed and resuspended in THY, made to 8% glycerol, aliquoted into 1-ml volumes, and stored at −80°C until used. The numbers of CFU per milliliter of these stocks were determined, after the aliquots had been frozen for 2 weeks, by plating a single quick-thawed diluted aliquot on 5% sheep’s blood TSA agar plates (Becton, Dickinson and Company). The calculated number of CFU was subsequently used to make dilutions for experiments from aliquots thawed at later times. In each experiment, the actual number of CFU administered was determined by plating on blood agar at the time of the assay. In the results, we demonstrate that the sequences of the αHD and PRD of PspA/WU2 expressed are identical to those of the αHD and PRD of PspA/A66.1 and the previously reported αHD and PRD sequences of PspA/A66 (60). The clade-defining region of PspA is the C-terminal region of about 100 amino acids of the αHD (28), which has been shown to be important in the cross-protection elicited by PspA (27, 38, 39).

### Human neutrophil purification.

Human neutrophils were purified from whole blood using Polymorphprep density gradient medium (Axis-Shield) as described previously (23).

### Ethics statement.

Blood procurement was conducted in accordance with institutional review board (IRB) protocol no. 150407007 ("Vaccine Potential of the Proline-Rich Domain of Pneumococcal Surface Protein A") approved by the University of Alabama at Birmingham IRB.

### Cloning of PspA/Rx1 and PspA/Rx1 fragments.

The construction of the seven 81- or 100-aa PspA/Rx1 fragments using the primers shown in Table 3 has been described previously (38). The cloned fragments 1 to 6 encode 100 amino acids. Fragment 7 encoded 81 amino acids. Each fragment had a 50-amino-acid overlap with adjacent fragments. The seven fragments covered aa −3 to 378 of the mature protein, including the αHD and PRD. The expressed rPspA/Rx1 (aa −18 to 372 of the mature protein) also included the αHD and PRD. These are shown diagrammatically in Fig. 1, where the numbering system is for the mature protein lacking its leader sequence.

**TABLE 3:** Primers used for generation of PspA fragments of PspA/Rx1

PspA fragment	Amino acids^*a*^	Primer sequence	Size of amplified DNA fragment (bp)
PspA/Rx1	−18 to 372	5′-TAGCTCGAGCCTCGAGATCTTAGGGGCTGGTTT-3′	1,185
		5′-TAGTTATCTAGATTTTGGTGCAGGAGCTGG-3′	
Fragment 1	−3 to 97	5′-CTCGAGGTAAGAGCAGAAGAATCTCCCGTA-3′	315
		5′-TTAGAATTCTATCATCTTATCTGCTGCGTCTTT-3′	
Fragment 2	48 to 147	5′-CTCGAGGAGGATCAGAAGAAAACTGAGGAG-3′	315
		5′-TTAGAATTCTTCTAGTTTTTTAGTAAGTTCTGG-3′	
Fragment 3	98 to 197	5′-CTCGAGGATGAAGCTAAGAAACGCGAAGAA-3′	315
		5′-TTAGAATTCCTCATCAATCTCTTTGAGCTCTTG-3′	
Fragment 4	148 to 247	5′-CTCGAGGAAGCTAAAGCAAAATTAGAAGAG-3′	315
		5′-TTAGAATTCAAGTTGATCTTCAAGTTTTGCAAT-3′	
Fragment 5	198 to 297	5′-CTCGAGTCTGAATCAGAAGATTATGCTAAA-3′	315
		5′-TTAGAATTCTTCTGGAGCTGGAGCTGGTTTTTC 3	
Fragment 6	248 to 347	5′-CTCGAGAAAGCTGCTGAAGAAAACAATAAT-3′	315
		5′-TTAGAATTCTGATCTACGAGCATAGTCTTCTTC-3′	
Fragment 7	298 to 378	5′-CTCGAGACTCCAGCCCCAGAAGCACCAGCT-3′	258
		5′-TTAGAATTCACCGTTTTCTTGTTTCCAGCCTGT-3′	

a Each fragment also contained nine non-PspA N-terminal amino acids (MHHHHHHLE) and four non-PspA C-terminal amino acids (EFEA).

### Expression and purification of PspA/Rx1 and fragments of PspA/Rx1.

PspA/Rx1 and PspA/Rx1 fragments were expressed in Escherichia coli (38). Protein expression was induced in mid-exponential-phase E. coli BL21-SI cultures by the addition of 300 mM NaCl. The recombinant proteins bearing N-terminal histidine tags were purified from the soluble fraction by affinity chromatography using Ni^2+^-charged resin (HisTrap HP column; GE Healthcare). Elution was carried out with 250 mM imidazole. The purified fractions were analyzed by sodium dodecyl sulfate (SDS)-polyacrylamide gel electrophoresis (PAGE), dialyzed (10 mM Tris-HCl [pH 8.0], 20 mM NaCl, 0.1% glycine), and stored at −20°C.

### Peptide arrays.

Peptide arrays of the αHD of PspA/Rx1 (PspA clade 2), composed of 15-amino-acid peptides containing 11-amino-acid overlaps, were obtained from CelluSpots, Intavis, Germany, and used as previously described (38).

### Monoclonal antibodies.

Table 1 lists the monoclonal antibodies used. MAbs Rx1MI003, -005, -006, and -007 were all produced for this study from spleen cells of BALB/cJ mice immunized with the αHD of PspA from strain Rx1 (family 1, clade 2) by the University of Alabama at Birmingham (UAB) Epitope Recognition and Immunoreagent Core Facility. All four of these antibodies were of the IgG1 isotype and reacted with the purified clade 2 PspA (Rx1) by Western blotting. The MAbs to PspA are specific for individual PspA epitopes, bind this epitope when it is expressed on diverse PspAs, and are generally not necessarily clade or family specific (28), but exceptions can occur (61). Using the dot blot assay (62) or Western blot assay (1), all four of these new MAbs reacted with the PspA in a lysate of the PspA family 1, clade 2, strain A66.1.

MAbs 8b2.19 and 1b2.21 (23) also reacted with PspA/A66.1 and were utilized in this study along with the MAb PR-1A4.7, which reacted with the NPB of the PRD of PspA/A66.1 (37). All three of these MAbs, when given passively, have been shown to protect mice from lethal challenge with strain A66.1 (23, 37).

All the antibodies were used as diluted ascitic fluid to avoid any potential denaturation that might result from the purification and concentration of the MAbs. Dilutions were in Ringer’s infection solution (Abbot Labs, Chicago, IL) as described previously (23). To determine the concentration of each antibody in the respective ascitic fluids, quantitative microzone electrophoresis was used to separate the protein components of the ascitic fluid on nitrocellulose (63). The protein bands were stained using Ponceau S red, and each band was subjected to densitometry. The total amount of protein contained in each ascitic fluid sample was determined using a Bio-Rad protein quantification assay. The amount of monoclonal antibody in each ascitic fluid sample was calculated by determining the fraction of the total densitometry of the scan that was contained in the monoclonal antibody band in the gamma globulin region and multiplying this fraction by the total number of micrograms of protein per milliliter in the ascitic fluid (64). MAb concentrations were expressed in micrograms per milliliter (63). This approach was based on the fact that in MAb-containing mouse ascitic fluid, the vast majority of immunoglobulin is comprised of the MAbs.

### Western blotting.

Recombinant proteins and fragments (4 μg) were separated by SDS-PAGE and transferred to a nitrocellulose membrane. Full-length PspA/Rx1 and PspA/Rx1 fragments were detected with the different monoclonal antibodies (10 μg/ml), MAbs 1b2.21, 8b2.19, Rx1MI003, Rx1MI005, Rx1MI006, and Rx1MI007, followed by incubation with anti-mouse IgG conjugated to horseradish peroxidase (HRP) (Sigma-Aldrich). Detection was performed using an enhanced chemiluminescence (ECL) kit (GE Healthcare).

### MSKA.

The modified surface killing assay (MSKA), based on an earlier report (65), was performed as described previously (23). Briefly, bacteria opsonized with MAbs and fresh mouse complement were distributed in 15-μl spots on the surface of blood agar plates. All spots were allowed to soak into the plate via air drying at room temperature. Next, 20 μl of a solution of human polymorphonuclear leukocytes was dispensed to completely cover each spot. The numbers of CFU were counted the next day, and data are expressed as the number of CFU relative to that for the no-antibody control. The MAbs were used as ascitic fluids diluted in Ringer’s injection solution rather than as isolated MAbs, which would have risked denaturing some MAbs more than others.

### Passive protection against lethal sepsis.

A passive immunization test was performed by injecting each mouse intraperitoneally (i.p.) with 100 μl of MAb-containing ascitic fluid prepared by dilution with Ringer’s injection solution to contain 5 μg of the MAb to be tested. Control mice received a 1/6 dilution of pooled normal mouse serum (NMS), which was adjusted to contain approximately the amount of total albumin in our diluted ascitic fluid samples. Each of the MAbs was tested in from 5 to 14 mice. The NMS control was given to 14 mice. Four hours later the mice were challenged intravenously (i.v.) with S. pneumoniae A66.1. Six- to 8-week-old female CBA/CaHN-Btk^xid^/J (CBA/N) mice (The Jackson Laboratory, Bar Harbor, ME) were challenged with approximately 450 CFU of A66.1. Infected mice were monitored every 6 h to determine the length of time until they were moribund. Each experiment ended after 14 days, which was more than a week after most mice became moribund. Mice were considered moribund when their body temperatures were 26°C or lower or they were not responsive to touch. The Mann-Whitney test was used to compare the time (in hours) to a moribund state for each MAb-immunized group with the time to a moribund state for the control group. All moribund mice and mice surviving for 14 days were euthanized by CO_2_ narcosis and subsequent cervical dislocation. The mice surviving for 14 days were assigned a time to a moribund state of 14.5 days when *P* values were calculated by the Mann-Whitney 2-sample rank test since they lived longer than any other mice in the experiment. All animal protocols were conducted in accordance with AAALAC guidelines approved by the UAB IACUC.
