# Supplementary material for: The Modified Surface Killing Assay Distinguishes between Protective and Nonprotective Antibodies to PspA
Source: mSphere. 2019 Dec 11;4(6):e00589-19. doi: 10.1128/mSphere.00589-19 (PMC6908419; doi:10.1128/mSphere.00589-19)
Supplement: TEXT S1 [file mSphere.00589-19-s0001.docx]

SUPPLEMENTAL TEXT 1

Supplemental Text 1: Strong structural similarities of PspA/Rx1, PspA/A66.1, and PspA/WU2. For historical reasons many of the mAb to PspA that were available for these studies had been made against PspA/Rx1. Strain Rx1 is a non-encapsulated mutant pneumococcus developed in the middle of the 20th century from D39 (1, 2). Rx1 and D39 have identical PspA sequences (3, 4). Two of the most frequent challenge strains used to test protective activity of mAb elicited by the Rx1 αHD, and other PspAs, have been capsular type 3 strains WU2, and A66.1. WU2 has been used in previous mapping studies of PspA epitopes (5). It is known that Rx1, A66.1 and WU2 are all PspA family 1, clade 2 (4, 6). A recent sequence of PspA/A66 (6) showed that its PspA was very similar to the known sequence for PspA/WU2 (4). For this study we re-sequenced both pspA/WU2 and the pspA of our sub-clone, A66.1, of strain A66. We observed that the mature PspA/A66.1 and PspA/WU2 sequences were both identical to that of the prior (6) PspA/A66 sequence. Moreover the αHDs of the PspA/A66, PspA/A66.1, and PspA/WU2 were identical with the PspA/D39 sequence for predicted amino acids 149 to 288 (position numbers refer to the PspA/Rx1/D39 mature sequence). The amino acid sequences of the αHDs PspA/A66, PspA/A66.1, and PspA/WU2 were 65.7% identical with PspA/Rx1 for amino acids in positions 18 through 148. Position 288 is the C-terminal amino acid of the αHD. The C-terminal 116 amino acids of the αHDs of the Rx1, WU2, and A66.1 PspAs comprises their clade-determining region (CDR) (4). The CDR has been shown previously (7-9).

SUPPLEMENTAL REFERENCES

1. Yother J, p 232-243, *in* Fischetti VA, Novick RP, Ferretti JJ, Portnoy DA, Rood JI, ed., *Gram-Positive Pathogens*, 2000

2. Shoemaker NB, Guild WR., *Mol Gen Genet* **128**:283-290, 1974.

3. Lanie JA, Ng WL, Kazmierczak KM, Andrzejewski TM, Davidsen TM, Wayne KJ, Tettelin H, Glass JI, Winkler ME. *J Bacteriol* **189**:38-51, 2007

4. Hollingshead SK, Becker R, Briles DE. *Infect Immun* **68**:5889-900, 2000.

5. McDaniel LS, Scott G, Kearney JF, Briles DE., *J Exp Med* **160**:386-397, 1984.

6. Hahn C, Harrison EM, Parkhill J, Holmes MA, Paterson GK., *Genome Announc 3*, 2015.

7. Vadesilho CF, Ferreira DM, Gordon SB, Briles DE, Moreno AT, Oliveira ML, Ho PL, Miyaji EN., *Clin Vaccine Immunol* **21**:940-8, 2014.

8. Roche H, Hakansson A, Hollingshead SK, Briles DE., *Infect Immun* **71**:1033-41, 2003.

9. McDaniel LS, Ralph BA, McDaniel DO, Briles DE., *Microb Pathog* 17:323-37, 1994.
