# Supplementary figures and images for: The Modified Surface Killing Assay Distinguishes between Protective and Nonprotective Antibodies to PspA
Source: mSphere. 2019 Dec 11;4(6):e00589-19. doi: 10.1128/mSphere.00589-19 (PMC6908419; doi:10.1128/mSphere.00589-19)

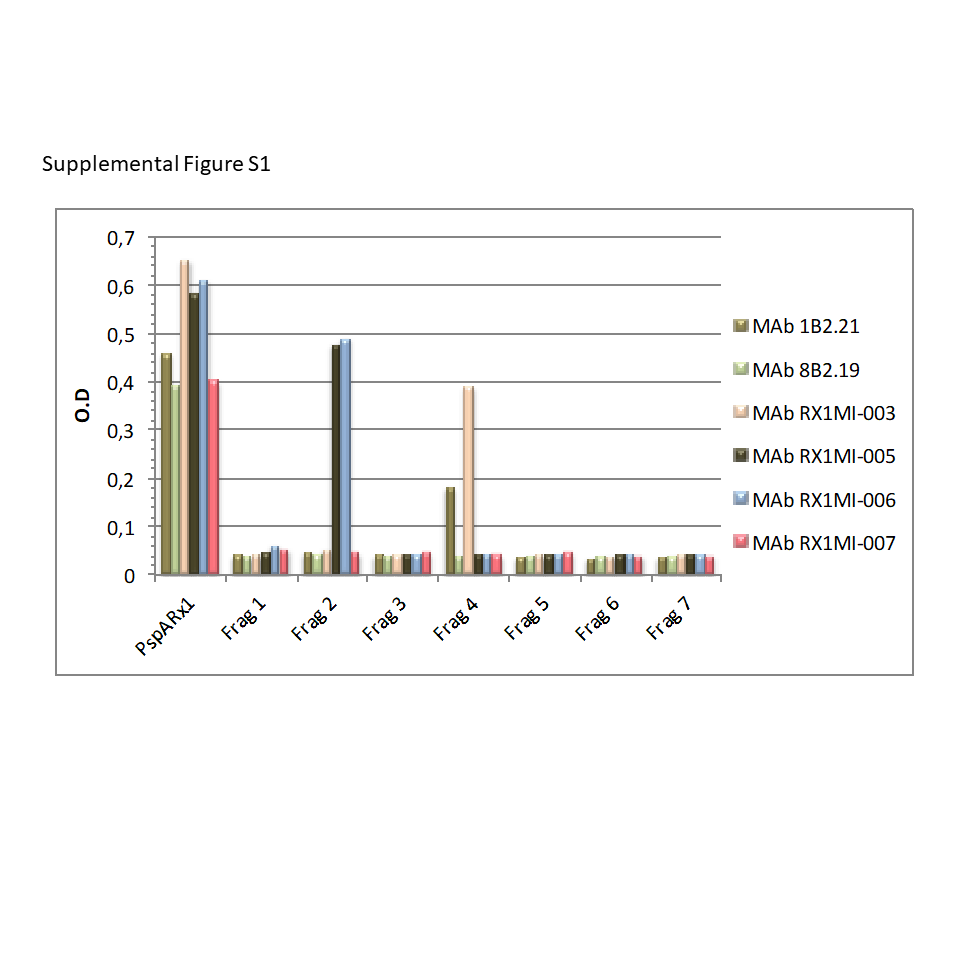

Supplement: FIG S1 [file mSphere.00589-19-sf001.tif]
